# Utilization of rehabilitation services among older adults with physical disabilities: the interactive influence of regional development and socioeconomic position

**DOI:** 10.3389/fpubh.2025.1535229

**Published:** 2025-06-04

**Authors:** Yi-Ran Wang, Xiao-Qian Yan, Fan Zhang, Chen-Tao Zhang, Wan-Nian Liang

**Affiliations:** ^1^Vanke School of Public Health, Tsinghua University, Beijing, China; ^2^Institute for Healthy China, Tsinghua University, Beijing, China; ^3^School of Nursing and Rehabilitation, Shandong University, Jinan, Shandong, China; ^4^School of Ecology and Environment, Renmin University of China, Beijing, China

**Keywords:** older adults, disability, socioeconomic status, rehabilitation services utilization, influence mechanism

## Abstract

**Background:**

The utilization of rehabilitation services by older adults with disabilities is an important health task in the context of population aging and disability expansion. This study aims to explore whether there are structural differences in the relationship between the socioeconomic position (SEP) of older adults with physical disabilities (PD) and their utilization of rehabilitation services across urban and rural areas.

**Methods:**

A total of 19,782 observations of older adults with PD from the 2007–2013 China National Survey on Disability and the 2006 Second National Sample Survey on Disability were included in this study. This study employs a multiplicative interaction effect model based on logistic regression to analyze the differential impact of individual SEP on the utilization of rehabilitation services among urban and rural older adults with PD.

**Results:**

The results indicate that higher annual per capita household income has a stronger influence on the utilization of rehabilitation services in urban areas (OR = 1.315, 95% CI 1.028–1.683). This may be due to the higher development level in urban areas, which amplifies the positive effects of high SEP on individual health investments. Conversely, individuals with lower SEP in urban areas may experience “self-limiting” behavior and difficulties in integrating into the urban and social welfare systems, further inhibiting the utilization of rehabilitation services.

**Conclusion:**

There is a structural urban–rural difference in the relationship between SEP and the utilization of rehabilitation services among older adults with PD. It is recommended to enhance both the accessibility and affordability of rehabilitation services for disadvantaged individuals in economically advantaged regions, while in economically disadvantaged regions, efforts should focus on improving the accessibility of rehabilitation infrastructure and strengthening the affordability of services for vulnerable populations. This can be achieved through legislative safeguards, financial assistance, and the development of a coordinated service delivery system.

## 1 Introduction

Aging is the most significant structural-demographic issue in the twenty-first century for China, and the health of the older adults is a critical challenge for the orderly development of an aging society. According to international consensus, a country or region is considered to have entered an aging society when the proportion of people aged 65 and over reaches 7% of the total population. Specifically, 7% marks the onset of a mildly aging society, 14% indicates a deeply aging society, and 20% signifies the transition to a severely aging society (1). Projections suggest that, even accounting for the impact of the two-child and three-child policies, China will continue to experience rapid population aging, with the country expected to enter a deeply aging society by 2022 and a severely aging society by 2033 (2). The aging of the disabled population is more severe, with the aging rate expected to rise from 57% in 2033 to 70% in 2050 (2). In terms of health status among older adults, it is predicted that the expected years of disability for individuals aged 60 and above in China will increase from 5.78 years in 2015 to 7.44 years in 2030 and 11.45 years in 2050 (2). The rise in expected years of disability is not solely due to population aging but also reflects complex interactions between demographic shifts, rising chronic disease burden and improvements in life expectancy. Studies suggest that while medical advancements extend lifespan, they may not fully offset disability risks, leading to longer periods of morbidity (3, 4).

This population health trend above, characterized by “rapid aging and disability expansion,” poses significant challenges to the supply and demand of rehabilitation services. Rehabilitation services are the core means to improve the functional status of disabled people. The characteristics of older adults with disabilities differ from those of younger disabled populations. This is because of the decline in the physiological functions of them, who have a higher incidence of geriatric diseases and disability rates. Therefore, they have greater needs for medical services, daily care, and relief support, and require more attention from society. China's rehabilitation policy framework has undergone significant development since the launch of the Healthy China 2030 initiative in 2016, which identified rehabilitation services as an essential component of healthcare (5). In 2017, the Regulations on Disability Prevention and Rehabilitation further reinforced this by mandating local governments to establish rehabilitation services networks (6). Supported by initiatives such as the Targeted Rehabilitation Services Program for Persons with Disabilities and the National Basic Public Services Equalization Plan, the country has made continuous progress in expanding rehabilitation services and has initially achieved broad coverage of basic rehabilitation provisions (7). Despite these advancements, there are still a large number of older adults with disabilities are unable to obtain rehabilitation assessment and referral services through institutionalized channels. By the end of 2019, ~36.817 million individuals in China had been officially certified as persons with disabilities (8), having undergone assessment by qualified institutions in accordance with relevant standards, such as the Classification and Grading Criteria of Disability for Persons with Disabilities (GB/T 26341-2010), and having lawfully obtained a disability certificate. Based on the estimated total of 108 million persons with disabilities in 2020 (2), the certification rate—that is, the share of disabled persons who have obtained official certification—was ~34.1%. This figure indicates that nearly two-thirds of the disabled population may remain outside the established mechanisms for disability identification, including screening, diagnosis, and evaluation, thereby impeding their integration into the rehabilitation services system (9). So the adequate utilization of rehabilitation services by older adults with disabilities is an important health task in the context of population aging and disability expansion.

Existing studies revealed Urban–rural disparities and socioeconomic position (SEP) pose significant challenges to rehabilitation access for China's aging population with disabilities. Individuals with higher income and education levels demonstrate significantly greater usage of rehabilitation services compared to their lower-income or less-educated counterparts (10). A substantial gap persists between the high demand and the low actual utilization of rehabilitation services among older adults with disabilities, particularly among those living in rural areas, with annual per capita household income below the national average, or with lower levels of education (11). Although national policies have expanded service coverage, substantial gaps in service utilization persist along socioeconomic lines. Numerous studies have demonstrated that older adults with disabilities who are socioeconomically disadvantaged face disproportionately greater obstacles in accessing rehabilitation services (9, 10). Additionally, the development of rehabilitation services remains uneven and insufficient across regions. The urban–rural divide among persons with disabilities is particularly pronounced, with marked inequalities in health outcomes resulting from unequal access to healthcare services (12). Compared to their urban counterparts, the rural older adults with disabilities have significantly limited access to both medical services and educational resources (13). Moreover, many rural households with disabled members fall into deep poverty due to the high financial burden of disability-related care (14). Older adults with disabilities in rural areas face compounded disadvantages: socioeconomic barriers such as lower income and education levels, and structural barriers stemming from systemic underinvestment in rural infrastructure. Lamy et al. (15) found in their research that there are differences in the utilization of ophthalmic examinations among diabetic patients in areas with different degrees of urbanization in the Midi-Pyrénées region of France. Liu et al. (16) pointed out that during the economic transformation period in China, the number of outpatient visits of urban and rural residents increased, but the number of rural residents using inpatient services relatively decreased. The place of residence affects service utilization, and the mechanisms involve factors such as service accessibility, geography, and transportation, as shown in relevant studies in the Americas, Asia, and Europe (17). Existing studies have laid a foundation, but there is still a need for in—depth research on the deep—seated mechanisms of the impact of regional development and optimizing medical resource supply to improve service utilization efficiency.

According to the theory of cumulative advantage, initial socioeconomic advantages are magnified over time through mechanisms of resource accumulation (18). In China, this dynamic manifests in systemic disparities wherein urban residents benefit from cumulative advantages in healthcare access, while rural residents experience a compounding of disadvantage. This study investigates how regional development levels interact with individual SEP to shape disparities in rehabilitation access. By integrating multidisciplinary theories with empirical analysis, it aims to explore the underlying causes of insufficient rehabilitation services utilization and the absence of motivating factors for service uptake. The study provides supplementary evidence to enhance understanding of the structural determinants of health equity among China's aging population.

## 2 Methods

### 2.1 Data source

The primary data for this study come from the 2007–2013 China Disabled Persons Status Monitoring Survey (hereinafter referred to as the monitoring survey), supplemented by the 2006 Second National Sampling Survey of Disabled Persons (hereinafter referred to as the second survey) and the China National Statistical Yearbook. Data on the utilization of rehabilitation services and SEP, individual conditions, and residential areas (urban and rural) are sourced from the monitoring survey and the second survey. Regional-level control variables primarily come from the National Statistical Yearbook, with regional economic level (provincial per capita GDP), regional education level (provincial average years of education), and regional medical level (number of medical and health technicians per thousand people) sourced from various editions of the China Statistical Yearbook and the China Health and Family Planning Statistical Yearbook.

**Figure 1:**
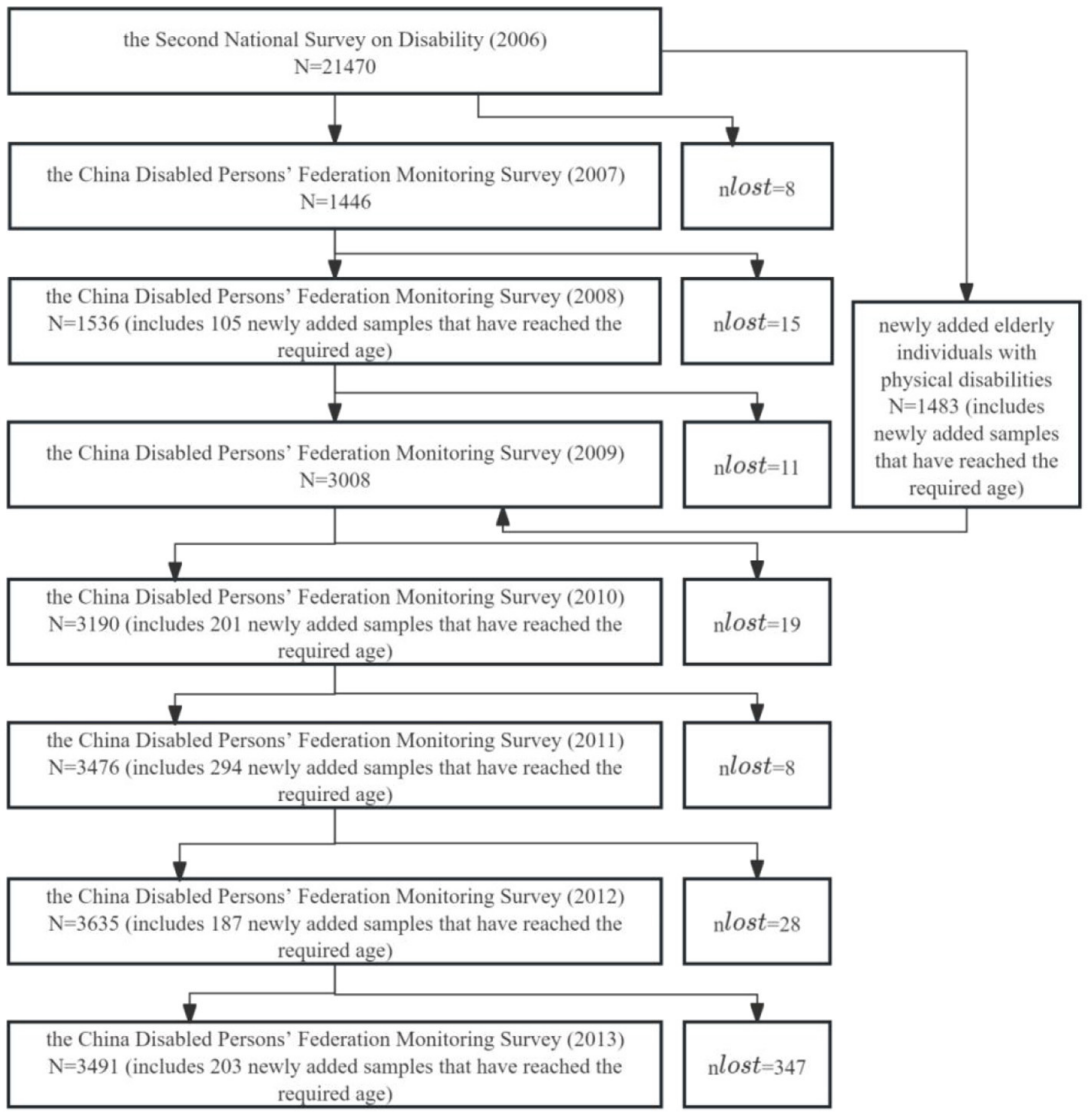
Sample details from 2007 to 2013.

The second survey's sample includes the population of all households at the survey time point, employing stratified, multi-stage, cluster, probability proportional sampling to obtain representative samples. A total of 734 counties (cities, districts), 2,980 townships (towns, streets), and 5,964 survey communities were selected, with an average of about 420 people per community. A total of 771,797 households and 2,526,145 individuals were surveyed, with a sampling ratio of 1.93‰ (19). The data from this survey are considered reliable (20). To timely understand changes in the status of disabled persons in China, the National Bureau of Statistics, Ministry of Civil Affairs, former Ministry of Health, and China Disabled Persons' Federation jointly launched the national monitoring survey on the status of disabled persons from 2007 to 2013 (21). The monitoring survey targets disabled persons randomly selected from the second national sampling survey of disabled persons conducted in 2006 (22). Specifically, the 2007 monitoring survey is based on the sample frame of the 2006 second survey, selecting one survey community from each of the 734 county-level samples as national monitoring sample units, and monitoring all disabled persons and their family conditions in these communities. In 2008, the 2007 samples were tracked, and in 2009, an additional survey community was added to each of the 734 counties (cities, districts), expanding from 734 to 1,467 communities. The 2009 samples were tracked in 2010, and in 2011, 12,724 new individuals were added to the existing disabled persons. The 2011 samples were tracked in 2012, and the 2012 samples were tracked in 2013. The main contents of the monitoring survey are based on the key indicators of the Well-off Index System for Disabled Persons in China and the main indicators of the second survey, covering aspects such as the life, rehabilitation, education, employment, community services, barrier-free environment, and legal services of disabled persons. As an extension of the 2006 second national sampling survey, the 2007–2013 national monitoring survey provides important information and data to timely, accurately, and comprehensively understand the status and changes of disabled persons (23). The 2007–2013 national monitoring data includes 19,782 observations of older adults with physical disabilities (PD), with annual sample details shown in Figure 1.

### 2.2 Study design and subjects

This study aims to explore whether there are structural differences in the relationship between the SEP of older adults with PD and their utilization of rehabilitation services across urban and rural areas. The study population comprises older adults aged 60 and above with simple PD, referred to as “older adults with PD” hereafter. PD refers to damage or dysfunction within the human locomotor system, resulting in limb deficiency, paralysis (paresis) of the limbs or trunk, deformities, and other related conditions. These impairments contribute to varying degrees of motor function loss and impose restrictions on daily activities and social participation. PD include: the absence, deformity, or functional impairment of the upper or lower limbs due to injury, illness, or developmental abnormalities; deformities or functional impairments of the spine resulting from injury, illness, or developmental abnormalities; and functional impairments of the trunk or limbs caused by injuries, diseases, or developmental abnormalities of the central and peripheral nerves (24). Simple PD refers to the condition of having a PD without the coexistence of other types of disabilities (including visual disability, hearing disability, speech disability, intellectual disability, and mental disability).

The dependent variable is whether the individual has used rehabilitation services, defined as having used rehabilitation treatment, training, or assistive devices during the monitoring period. Key independent variables include: (1) Education level: Based on the era characteristics of the respondents' education stages, the individual education levels in the questionnaire were integrated and categorized into no formal education, basic education (including primary and junior high school), and high school and above education (including high school, technical secondary school, and university and above); (2) Income level: The household's total income during the monitoring period (including wage annual income, urban operational annual net income, rural operational annual gross income, property annual income, transfer annual income, sales of property annual income, and loan annual income) divided by the household population to obtain the per capita annual income, categorized into high, medium, and low based on annual tertiles. Control variables are categorized into: (1) Individual-level variables: These include disability level, gender, age, marital status, and basic medical insurance coverage. The disability level of individuals with PD was determined based on the classification and grading standards in the 2006 second national sampling survey and the “Classification and Grading of Disabilities” (GB/T26341-2010). According to existing studies (25), disability levels 1–4 were categorized into mild disability (level 4) and moderate to severe disability (levels 1–3). Age was divided into young-old (60–69 years), middle-old (70–79 years) and oldest-old (80 years and above). Marital status was categorized into married (including first marriage and remarriage with a spouse) and unmarried, divorced, or widowed (combined as without a spouse). Basic medical insurance includes New Rural Cooperative Medical System, Urban Employee Basic Medical Insurance, Urban Resident Basic Medical Insurance, and Basic Medical Insurance for Self-employed Individuals. (2) Regional-level variables: These include residence areas (including rural residence area and urban residence area), regional economic level (measured by provincial per capita GDP), regional education level (measured by provincial average years of education), and regional medical level (measured by the number of medical technicians per thousand people). Given the monitoring period spans 2 years, this study uniformly uses the provincial per capita GDP, provincial average years of education, and the number of medical technicians per thousand people from the later year, classified as high or low levels based on a comparison with the annual average values. (3) Time fixed effects (26).

### 2.3 Statistical analysis

First, a descriptive analysis was conducted on the utilization rate of rehabilitation services among urban and rural older adults, and chi-square tests were employed to assess group differences. Cross-tabulated stratified descriptions were performed for groups with different SEP in urban and rural areas. Specifically, the study's core explanatory variables (education level and per capita annual household income) were cross-tabulated with urban–rural residence, and stratified odds ratio (OR) homogeneity tests and stratified chi-square tests were used to assess the significance of inter-stratum differences. This aimed to determine whether urban–rural residence potentially affects the association between the core explanatory variables (education level and per capita annual household income) and the outcome variable (rehabilitation services utilization), thereby providing statistical evidence for the necessity of subsequent heterogeneity analysis. The Breslow-Day method was employed to test the homogeneity of stratified ORs, i.e., to examine the consistency of ORs across different strata. If significant differences in ORs between strata were observed, it would indicate from a statistical perspective that heterogeneity analysis between the core explanatory variables and the outcome variable is necessary. In this case, ORs should not be pooled, and stratified reporting is recommended.

Second, the association analysis was conducted using logistic regression models. In the section on interaction effect analysis, this study first presents graphical results to preliminarily explore potential differences between groups. Binary logistic regression was used to report the predicted probability of each interaction group, holding all variables except the main variables and interaction terms at their mean values.

Third, the multiplicative interaction effect method based on logistic regression models was employed to rigorously calculate and analyze the interaction effect of urban–rural residence and SEP on the utilization of rehabilitation services. The calculation for the multiplicative interaction effect is as follow.


$$\begin{array}{l} \frac{\frac{p_{x=1,\;z=1}}{{1-p}_{x=1,\;z=1}}/\frac{p_{x=1,\;z=0}}{{1-p}_{x=1,\;z=0}}}{\frac{p_{x=0,\;z=1}}{{1-p}_{x=0,\;z=1}}/\frac{p_{x=0,\;z=0}}{{1-p}_{z=0,\;z=0}}} \end{array}$$

Taking the interaction between residence area and income level as an example, the multiplicative interaction effect can be interpreted as the ratio of the odds ratio (OR_ab_) of rehabilitation services utilization between high- and low-income groups in urban areas, to the OR_cd_ between high- and low-income groups in rural areas.


$$\begin{array}{l} \frac{OR_{ab}}{OR_{cd}} \end{array}$$

**Figure 2:**
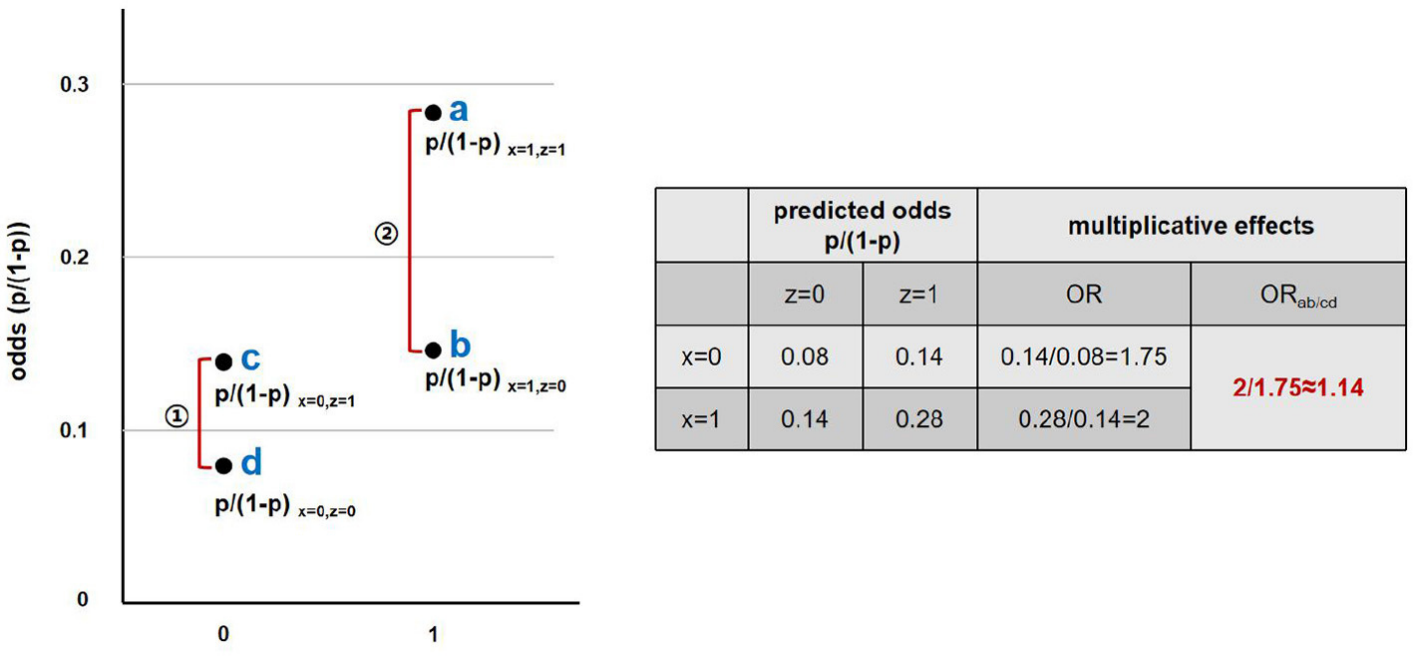
Illustration of the multiplicative interaction effect method.

This ratio reflects whether the effect of income on rehabilitation services utilization varies across residence areas, i.e., whether a significant interaction exists between income level and place of residence. An illustration of the multiplicative interaction effect method is shown in Figure 2 (data are hypothetical).

All statistical analyses in this study were conducted using Stata 16.0. The significance levels were set at 0.1, 0.05, and 0.01. Clustered standard errors were used to adjust for errors caused by repeated observations of the same sample over multiple years.

## 3 Results

### 3.1 Urban–rural differences in rehabilitation services utilization by different SEP among older adults with PD

The utilization rate of rehabilitation services among urban older adults with PD (54.42%) is generally higher than that of their rural counterparts (39.68%), with a statistically significant difference between the two.

In terms of income level, within each income group, the utilization rate of rehabilitation services among urban older adults with PD is higher than that in rural areas. Regardless of residence in urban or rural areas, a trend is observed whereby higher income groups tend to have higher utilization rates of rehabilitation services. Specifically, in urban areas, the utilization rates for the middle- and high-income groups are 9.47 and 20.95 percentage points higher, respectively, than that of the low-income group. In rural areas, the rates for the middle- and high-income groups are 7.45 and 14.02 percentage points higher, respectively, than that of the low-income group. The disparities are more pronounced in urban areas (Table 1).

**Table 1:** Urban–rural differences in rehabilitation services utilization by SEP among older adults with PD.

**Category**	**Rehabilitation services utilization rate (%)**	**Chi-square test**	***P***	**Rehabilitation services utilization rate by educational level (%)**			**Rehabilitation services utilization rate by income level (%)**		
				**No formal education**	**Basic education**	**High school and above**	**Low**	**Medium**	**High**
Rural	39.68	359.7582	< 0.01	40.49	38.46	48.94	33.73	41.18	47.75
Urban	54.42			50.71	53.93	61.97	39.65	49.12	60.60

Regarding education level, within each education group, the utilization rate of rehabilitation services among urban older adults with PD is also higher than that in rural areas. Regardless of residence in urban or rural areas, there is a general trend where groups with higher education levels tend to have higher utilization rates of rehabilitation services. In rural areas, the utilization rate among those with basic education is slightly lower than that of the group with no formal education, but the difference is minimal. Therefore, overall, the trend that “groups with higher education levels tend to have higher utilization rates of rehabilitation services” still holds. Specifically, in rural areas, the utilization rate among those with high school education or above is 8.45 percentage points higher than that of the group with no formal education. In urban areas, the rates for those with basic education and high school education or above are 3.22 and 11.26 percentage points higher, respectively, than that of the group with no formal education. Again, the disparity is more significant in urban areas (Table 1).

The results of the Breslow-Day test show that the relationship between income and rehabilitation services utilization differs significantly between urban and rural areas (χ^2^ = 21.45, *P* < 0.01), indicating a significant interaction effect between urban–rural residence and income on rehabilitation services utilization. Similarly, the relationship between education level and rehabilitation services utilization also shows a statistically significant difference between urban and rural areas (χ^2^ = 13.66, *P* < 0.01), indicating an interaction effect between urban–rural residence and education level on rehabilitation services utilization (Table 2).

**Table 2:** Significance test of the interaction effects between SEP and urban–rural residence.

**Heterogeneity stratification variable**	**Significance of interaction between income and urban–rural residence**	***P***	**Significance of interaction between education level and urban–rural residence**	***P***
Urban–rural residence area	21.45	< 0.01	13.66	< 0.01

### 3.2 Urban–rural differences in the impact of SEP on rehabilitation services utilization among older adults with PD

#### 3.2.1 Urban–rural differences in the impact of income levels on rehabilitation services utilization among older adults with PD

**Figure 3:**
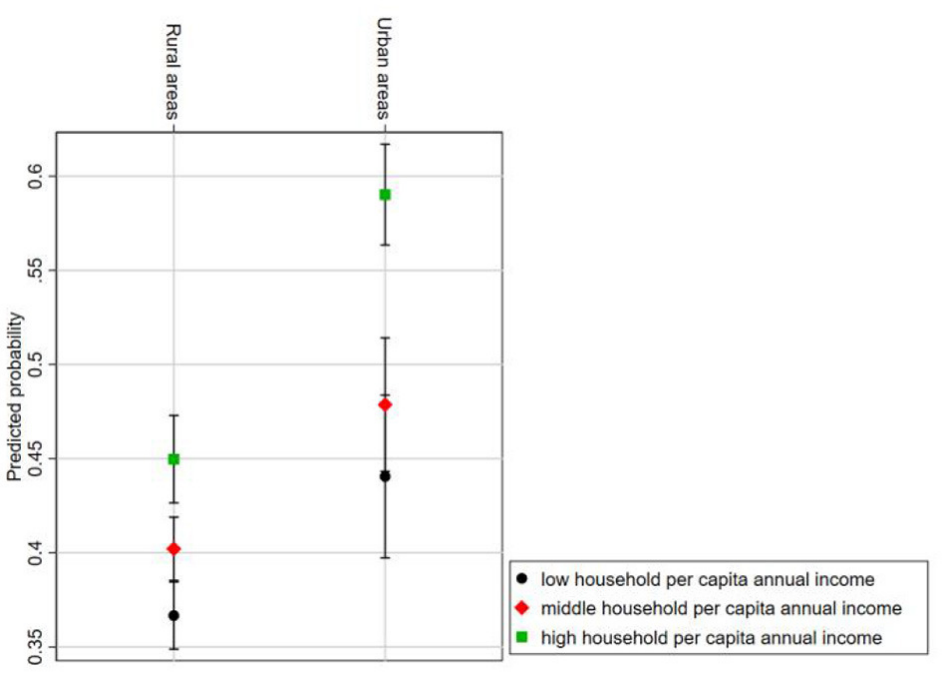
Predicted probability of rehabilitation services utilization by income level and urban–rural interaction group.

Figure 3 presents the predicted probability of different income levels on the utilization of rehabilitation services within urban and rural older adults with PD. The relative sizes of the predicted probabilities for each income level indicate that urban older adults exhibit higher probabilities of utilizing rehabilitation services compared to their rural counterparts across all income levels. This study will further employ interaction effects testing to explore the differential impact of income levels on rehabilitation services utilization between urban and rural older adults with PD.

Table 3 shows the differential impact of annual per capita household income on the utilization of rehabilitation services among older adults with PD in China across different urban and rural residence groups, using rural residents as the reference group. The results indicate that medium household per capita income does not significantly differ between urban and rural older adults in terms of impact on rehabilitation services utilization. However, high household per capita income has a significantly greater impact on the utilization of rehabilitation services among urban older adults compared to their rural counterparts (OR = 1.315, 95% CI 1.028–1.683), indicating that high-income urban older adults are ~32% more likely to use rehabilitation services than their high-income rural counterparts.

**Table 3:** Urban–rural differences in the impact of household per capita income on rehabilitation services utilization among older adults with PD.

**Variables**	**OR**	**S.E**.	***z***	**95% CI**	***P***
**Interaction items (Residence areas** × **Household per capita**					
**income levels)** ^*^					
Rural × low income	1	–	–	–	–
Rural × medium income	1	–	–	–	–
Rural × high income	1	–	–	–	–
Urban × low income	1	–	–	–	–
Urban × medium income	1.004	0.119	0.04	0.796–1.268	=0.970
Urban × high income	1.315	0.165	2.18	1.028–1.683	< 0.05
**Residence area**					
Rural area	1	–	–	–	–
Urban area	1.383	0.142	3.16	1.131–1.692	< 0.01
**Household per capita income**					
Low	1	–	–	–	–
Medium	1.171	0.058	3.18	1.062–1.291	< 0.01
High	1.439	0.092	5.69	1.269–1.631	< 0.01
**Education level**					
No formal education	1	–	–	–	–
Basic education	0.993	0.049	−0.15	0.901–1.093	=0.880
High school and above education	1.232	0.133	1.93	0.996–1.522	< 0.1
**Disability level**					
Mild disability	1	–	–	–	–
Moderate to severe disability	1.804	0.092	11.55	1.632–1.994	< 0.01
**Gender**					
Female	1	–	–	–	–
Male	1.000	0.049	0.00	0.908–1.102	=0.998
**Age**					
Young-old individuals	1	–	–	–	–
Middle-old individuals	1.440	0.069	7.62	1.311–1.581	< 0.01
Oldest-old individuals	1.792	0.125	8.39	1.564–2.054	< 0.01
**Marital status**					
Unmarried	1	–	–	–	–
Married	1.076	0.055	1.44	0.974–1.188	=0.149
**Basic medical insurance coverage**					
No	1	–	–	–	–
Yes	1.194	0.084	2.53	1.041–1.371	< 0.05
**Regional economic level**					
Low level	1	–	–	–	–
High level	1.201	0.067	3.26	1.076–1.340	< 0.01
**Regional medical level**					
Low level	1	–	–	–	–
High level	0.727	0.042	−5.52	0.650–0.814	< 0.01
**Regional education level**					
Low level	1	–	–	–	–
High level	0.991	0.053	−0.16	0.893–1.100	=0.871
**Year**					
2007	1	–	–	–	–
2008	0.914	0.058	−1.43	0.808–1.034	=0.153
2009	1.021	0.065	0.33	0.902–1.156	=0.745
2010	1.278	0.081	3.87	1.128–1.447	< 0.01
2011	1.461	0.095	5.80	1.285–1.660	< 0.01
2012	1.933	0.127	10.04	1.699–2.198	< 0.01
2013	2.022	0.133	10.66	1.776–2.301	< 0.01

OR, odds ratio; CI, confidence intervals.

^*^For within-group comparisons, the low-income subgroup served as the reference category in both urban and rural groups.

#### 3.2.2 Urban–rural differences in the impact of educational levels on rehabilitation services utilization among older adults with PD

**Figure 4:**
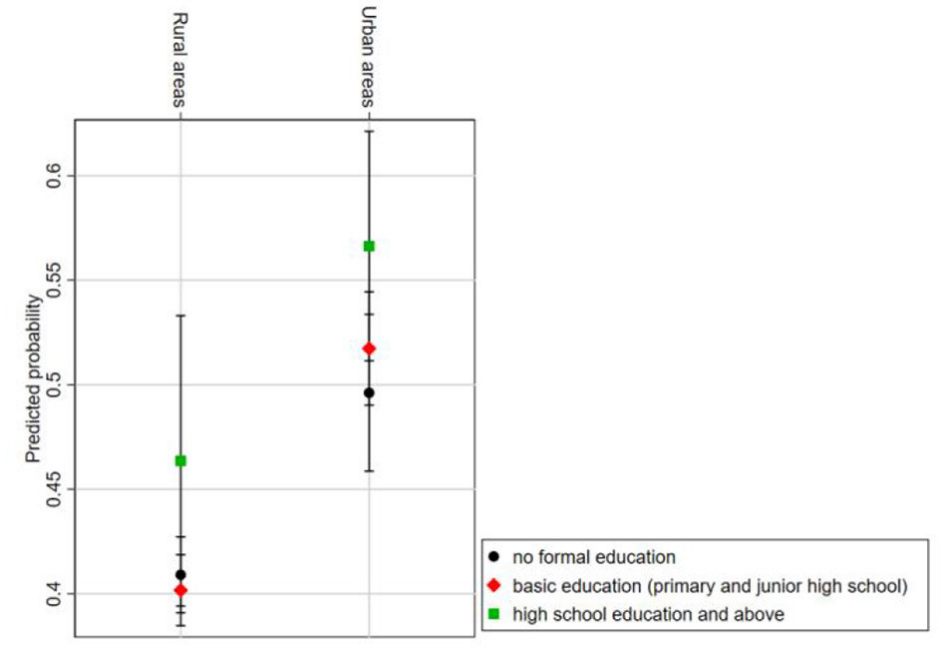
Predicted probability of rehabilitation services utilization by educational level and urban–rural interaction group.

Figure 4 presents the predicted probability of different educational levels on the utilization of rehabilitation services within urban and rural older adults with PD. The relative sizes of the predicted probabilities indicate that urban older adults exhibit higher probabilities of utilizing rehabilitation services compared to their rural counterparts across all educational levels. This study will further employ interaction effects testing to explore the differential impact of educational levels on rehabilitation services utilization between urban and rural older adults with PD.

Table 4 shows the differential impact of educational level on the utilization of rehabilitation services among older adults with PD in China across different urban–rural residency status groups, using rural areas as the reference group. The results indicate that, when using “no formal education” as the reference category within groups, the impact of basic education and higher education on the utilization of rehabilitation services among older adults with PD in urban areas does not significantly differ from their impact in rural areas.

**Table 4:** Urban–rural differences in the impact of educational levels on rehabilitation services utilization among older adults with PD.

**Variables**	**OR**	**S.E**.	***z***	**95% CI**	***P***
**Interaction items (Residence areas** × Educational levels)^*^					
Rural × no formal education	1	–	–	–	–
Rural × basic education	1	–	–	–	–
Rural × high school and above education	1	–	–	–	–
Urban × no formal education	1	–	–	–	–
Urban × basic education	1.130	0.122	1.14	0.916–1.396	=0.254
Urban × high school and above education	1.066	0.225	0.30	0.706–1.611	=0.761
**Residence area**					
Rural area	1	–	–	–	–
Urban area	1.454	0.130	4.17	1.219–1.734	< 0.01
**Education level**					
No formal education	1	–	–	–	–
Basic education	0.968	0.053	−0.60	0.870–1.077	=0.552
High school and above education	1.266	0.200	1.49	0.928–1.726	=0.136
**Household per capita income**					
Low	1	–	–	–	–
Medium	1.165	0.053	3.35	1.066–1.274	< 0.01
High	1.575	0.088	8.12	1.411–1.757	< 0.01
**Disability level**					
Mild disability	1	–	–	–	–
Moderate to severe disability	1.806	0.092	11.56	1.634–1.997	< 0.01
**Gender**					
Female	1	–	–	–	–
Male	0.996	0.049	−0.08	0.904–1.098	=0.939
**Age**					
Young-old individuals	1	–	–	–	–
Middle-old individuals	1.447	0.069	7.71	1.317–1.589	< 0.01
Oldest-old individuals	1.804	0.125	8.49	1.574–2.067	< 0.01
**Marital status**					
Unmarried	1	–	–	–	–
Married	1.074	0.054	1.40	0.972–1.186	=0.162
**Basic medical insurance coverage**					
No	1	–	–	–	–
Yes	1.214	0.085	2.76	1.058–1.394	< 0.01
**Regional economic level**					
Low level	1	–	–	–	–
High level	1.195	0.067	3.19	1.071–1.334	< 0.01
**Regional medical level**					
Low level	1	–	–	–	–
High level	0.732	0.042	−5.43	0.654–0.819	< 0.01
**Regional education level**					
Low level	1	–	–	–	–
High level	0.994	0.053	−0.12	0.896–1.103	=0.908
**Year**					
2007	1	–	–	–	–
2008	0.912	0.057	−1.46	0.806–1.032	=0.144
2009	1.016	0.064	0.25	0.897–1.151	=0.800
2010	1.272	0.081	3.79	1.123–1.440	< 0.01
2011	1.454	0.095	5.73	1.279–1.653	< 0.01
2012	1.923	0.126	9.96	1.691–2.187	< 0.01
2013	2.011	0.133	10.58	1.767–2.289	< 0.01

OR, odds ratio; CI, confidence intervals.

^*^For within-group comparisons, the subgroup with no formal education served as the reference category in both urban and rural groups.

## 4 Discussion

From the perspective of income level, compared to low household per capita income, high household per capita income has a stronger positive effect on the utilization of rehabilitation services among older adults with PD in urban areas than in rural areas. The distribution of rehabilitation services utilization probabilities across different income and urban–rural groups shows that this effect primarily arises from the higher and more significantly increased probability of rehabilitation services utilization among high-income older adults with PD in urban areas compared to their high-income rural counterparts. In contrast, low-income older adults with PD in urban areas have a higher probability of using rehabilitation services compared to their low-income rural counterparts, but the increase is smaller. Essentially, this reflects a more pronounced socioeconomic inequality in rehabilitation services utilization among older adults with PD in urban areas, indicating a pro-rich bias in urban rehabilitation services utilization.

The theory of cumulative advantage suggests that the convergence of multiple advantageous resources can amplify the effect of these advantages (27). Under the long-term influence of the urban–rural dual structure, basic public services and resources have been biased toward urban residents, while rural areas have lagged significantly in terms of public resources and basic public services (28). Wealthier individuals have relatively higher economic capacity, awareness, and social influence. The concentration of resources in urban areas further enhances the ability and effectiveness of wealthy groups in acquiring resources. Conversely, the cumulative disadvantage effect is evident among the least affluent groups in rural areas (29). Low-income older adults with PD face lower economic capacity, coupled with limited health resources and medical standards in rural areas, which may result in a more severe lack of rehabilitation services utilization—an issue that underscores the importance of spatial equity in service distribution, as emphasized by Witten et al. (30), whose work on urban environmental quality and community resources highlights the critical role of infrastructure and geographic access in shaping welfare. Since the United Nations Convention on the Rights of Persons with Disabilities came into effect in 2008, China has attached great importance to establishing public infrastructure and service support systems for persons with disabilities (31). However, due to significant regional disparities in economic development and government capacity, there are also regional differences in public infrastructure and services for persons with disabilities (32). The number of health technicians per 1,000 population in urban areas is significantly higher than in rural areas, and the number of hospital beds in urban medical institutions is 2.2 times that in rural areas (32). These disparities directly affect rural persons with disabilities in terms of access to and quality of rehabilitation services (33). Improving the spatial distribution of public facilities for persons with disabilities is of crucial significance for enhancing their wellbeing, achieving sustainable development, and promoting social equity (30). For relatively affluent older adults with PD in rural areas, despite facing similar issues of limited health resources and medical standards, their higher economic capacity allows them to benefit from the limited local rehabilitation resources or seek resources elsewhere. This method of compensating for the shortcomings of disadvantageous resources through advantageous resources can be attributed to the resource substitution theory (27).

In contrast, low-income older adults with PD in urban areas face weaker positive effects of urban factors on rehabilitation services utilization compared to high-income groups. First, as a form of consumption behavior, rehabilitation services utilization inequality is rooted in the wealth gap among urban residents. Since 2006, the wealth gap within urban areas in China has widened, with inequitable capital accumulation further exacerbating wealth disparity. Economic fluctuations have had a polarized impact on different income groups (34). The increasing wealth gap amplifies the disparity in consumption behavior between high- and low-income groups. Additionally, the siphoning effect of affluent regions attracts investment, further consolidating and promoting their advantages (35), providing strong external support and advantageous channels for rehabilitation services utilization among older adults with PD in these regions. Over time, the monopolization of health resources by high-income groups in urban areas may lead to a “stable difference” in health levels between high- and low-income groups, or even a Matthew effect, where the wealthy become healthier and the poor become more vulnerable (36), leading to a vicious cycle of “disability, poverty, and service deficiency.” Unlike their affluent rural counterparts, low-income older adults with PD in urban areas, despite residing in resource-rich urban regions, face self-limiting development due to low income, making it difficult for them to achieve rehabilitation services utilization through resource substitution. From the perspective of social integration, due to influences from resources, awareness, and capabilities, low-income groups—especially low-income migrants without urban residency—have a significantly lower level of social integration than high-income groups. Moreover, the positive relationship between social integration and the utilization of basic public health services is weaker among low-income groups than among high-income groups, displaying a “double disadvantage” in their efforts to achieve “urbanization” and access their entitled social benefits (37). Some empirical studies also show that the health behavior performance of disadvantaged groups in the most developed areas does not significantly differ from that of groups in less developed areas (15).

## 5 Limitations

This study contains individual-level time-invariant variables (educational attainment) among the core explanatory variables. Therefore, a bidirectional fixed effects model controlling for time-fixed effects and individual fixed effects was not utilized. Instead, logistic regression analysis with time-fixed effects and provincial control variables was employed. This approach may somewhat weaken the model's control over omitted variable bias.

## 6 Conclusions

There exists a structural urban–rural disparity in the relationship between SEP and the utilization of rehabilitation services among older adults with PD. The 14th Five-Year Plan for the Development of National Aging Undertakings and the Older Adults Care Service System explicitly calls for “narrowing the urban–rural gap in care services for older adults” and “improving the accessibility of rehabilitation nursing services.” The study's findings—namely the *pro-rich bias* in urban rehabilitation services utilization and the resource scarcity in rural areas—highlight urgent challenges that must be addressed in order to achieve these policy goals.

To this end, it is recommended to enhance both the accessibility and affordability of rehabilitation services for disadvantaged individuals in economically advantaged regions, while in economically disadvantaged regions, efforts should focus on improving the accessibility of rehabilitation infrastructure and strengthening the affordability of services for vulnerable populations. This can be achieved through legislative safeguards, financial assistance, and the development of a coordinated service delivery system. First, the efforts should be intensified to provide inclusive medical and rehabilitation services for older adults with disabilities, ensuring access to continuous and integrated care that includes treatment, rehabilitation, and nursing support. Second, the implementation of a “basic older adults care service list” system, as proposed in the 14th Five-Year Plan, should be advanced. This system should incorporate essential rehabilitation services—such as functional training and assistive device fitting—into targeted subsidy programs for recipients of urban subsistence allowances and low-income households. Third, it is also essential to strengthen county-level rehabilitation service capacity. Under the guideline “Enhancing the Capacity of Rehabilitation Medical Services” outlined in the Opinions on Accelerating the Development of Rehabilitation Medical Services, primary-level institutions should enhance their rehabilitation service capabilities and improve the overall capacity of rehabilitation medical services at the grassroots level. Additionally, efforts should be made to advance the thorough implementation of the Regulations on Rehabilitation Services for Persons with Disabilities, which define the legal status of rehabilitation services as part of basic public services. A national unified “Rehabilitation Needs Assessment Standard for Older Adults with Disabilities” should also be established to ensure consistency in service delivery and eligibility assessments. Finally, it is recommended to strengthen the disability reporting and registration system in disadvantaged regions. Leveraging the existing basic public health service network and using standardized disability screening tools, this system can collect detailed data on disability incidence and risk. Such a framework would not only expand coverage of the disabled population but also enable the early identification of disabling diseases and injuries, thereby facilitating more timely and effective rehabilitation interventions.

## Data Availability

The data analyzed in this study is subject to the following licenses/restrictions: The data that support the findings of this study are available from the Institute of Population Research of Peking University (IPR). Data are available with the permission of IPR. Requests to access these datasets should be directed to 00-86-010-62751974.
